# Modulated Start-Up Mode of Cancer Cell Migration Through Spinophilin-Tubular Networks

**DOI:** 10.3389/fcell.2021.652791

**Published:** 2021-03-09

**Authors:** Soyoung Hwang, Peter Chang-Whan Lee, Dong Min Shin, Jeong Hee Hong

**Affiliations:** ^1^Department of Physiology, College of Medicine, Gachon University, Incheon, South Korea; ^2^Department of Biomedical Sciences, University of Ulsan College of Medicine, Asan Medical Center, Seoul, South Korea; ^3^Department of Oral Biology, Yonsei University College of Dentistry, Seoul, South Korea; ^4^Department of Health Sciences and Technology, GAIHST, Lee Gil Ya Cancer and Diabetes Institute, Incheon, South Korea

**Keywords:** spinophilin, migration, tight junction, tubular network, bicarbonate transporter

## Abstract

Spinophilin (SPL) is a multifunctional actin-binding scaffolding protein. Although increased research on SPL in cancer biology has revealed a tumor suppressive role, its modulation in cancer biology, and oncological relevance remains elusive. Thus, we determined the role of SPL in the modulation of the junctional network and cellular migration in A549 lung cancer cell line. Knockdown of SPL promoted cancer cell invasion in agarose spot and scratch wound assays. Attenuation of SPL expression also enhanced invadopodia, as revealed by enhanced vinculin spots, and enhanced sodium bicarbonate cotransporter NBC activity without enhancing membranous expression of NBCn1. Disruption of the tubular structure with nocodazole treatment revealed enhanced SPL expression and reduced NBC activity and A549 migration. SPL-mediated junctional modulation and tubular stability affected bicarbonate transporter activity in A549 cells. The junctional modulatory function of SPL in start-up migration, such as remodeling of tight junctions, enhanced invadopodia, and increased NBC activity, revealed here would support fundamental research and the development of an initial target against lung cancer cell migration.

## Introduction

Spinophilin (SPL) is an actin-binding multifunctional protein that regulates protein interaction, enhances the activity of transporters, and modulates the structure of the cytoskeleton, which is involved in scaffolding, to modulate synaptic plasticity (Schuler and Peti, 2008). SPL has been reported to interact with F-actin, protein phosphatase 1, and zona occludens (ZO)-1 (Sarrouilhe et al., 2006). SPL plays a critical role in the nervous system, including in the regulation of dendritic spine function, synaptic plasticity, and neuronal cell migration (Allen et al., 1997; MacMillan et al., 1999; Ferrer et al., 2011). More recently, deletion of SPL was shown to promote cancer cell proliferation by phosphorylation of p53, demonstrating a new role in tumor suppression (Ferrer et al., 2011). Reduced SPL levels have been revealed in a subset of human lung tumors (Molina-Pinelo et al., 2011). In addition, Schwarzenbacher et al. (2015) addressed that SPL-silenced cancer xenografts showed the increased metastatic potential features. The SPL-associated tight junction protein ZO-1 is known as a junctional modulator involved in cell-cell adhesion, barrier formation, and cellular migration (Tornavaca et al., 2015). Cellular adhesion and junctional systems are required to maintain mechanical stability (Porterfield and Prescher, 2015), and modulation of mechanical stability is a typical feature of cancer cell migration. Although increased research on the role of SPL in cancer biology has revealed a tumor-suppressive role, its modulation in cancer and its oncological relevance to junctional interactive proteins such as ZO-1 remain unclear. Including the role as a scaffolding protein, the promiscuous molecular role of SPL should be identified the direct mechanisms in stages.

Cancer metabolism generates acidic metabolic products, including lactic acid, carbon dioxide, and protons (Sedlakova et al., 2014). Excessive production of acidic metabolites by cancer cells under hypoxic conditions makes the tumor microenvironment more acidic than that of normal tissue (Sedlakova et al., 2014). To drive more acid-producing pathways, cancer cells activate the buffering pH machinery that regulates intracellular pH away from the acidity of the microenvironment to drive transmembrane-associated ion flux through ion transporters and exchangers. The movement of ions through transporters is related to cellular movement. Thus, the role of various transporters is also important in cancer development and cancer cell migration (Schwab, 2001; Chen et al., 2007; Schwab and Stock, 2014; Stock and Schwab, 2015). More recently, the sodium-bicarbonate cotransporter NBCn1 was revealed as the migratory machinery in migratory lung cancer cells (Hwang et al., 2020).

In this study, we determined the role of SPL in the modulation of junctional network, cellular migration, and associated migratory machinery. The investigation of junctional modulatory function of SPL and migratory modification by functional inhibitors of SPL can help in the development of a potential target against lung cancer cell migration.

## Materials and Methods

### Reagents and Plasmids

β-Actin antibody (A3854), nocodazole (M1404), 4,4′-diisothiocyanatostilbene-2,2′disulfonate (DIDS, D3514), and cytochalasin D (C8273) were purchased from Sigma (Saint-Louis, MO, United States). Vinculin (ab73412), CD73 (ab175396), NBCn1 (ab82335), tubulin (ab56676), N-cadherin (ab76011), E-cadherin (ab15148), and vimentin (ab92547) were purchased from Abcam (Cambridge, MA, United States). ZO-1 antibody (67–7300) was purchased from Thermo Fisher (Waltham, MA, United States). 2′,7′-Bis-(2-carboxyethyl)-5-(and-6)-carboxyfluorescein-(BCECF)-AM was purchased from TEFlabs (0061, Austin, TX, United States). Alexa Fluor^TM^ 488 phalloidin (A12379) and pluronic acid (F-127, 20% in dimethyl sulfoxide, P3000MP) were purchased from Invitrogen (Carlsbad, CA, United States). SPL antibody (AB5669) was purchased from Millipore (Germany). The GFP-tagged human SPL construct was kindly provided by Dr. Shmuel Muallem (National Institute of Dental and Craniofacial Research, National Institutes of Health, Bethesda).

### Cell Culture

The lung adenocarcinoma cell line A549 was obtained from the American Type Culture Collection (Rockville, MD, United States, CRM-CCL-185) and maintained in Roswell Park Memorial Institute (RPMI) 1640 medium (11875093, Invitrogen) containing 10% FBS (1600–044, Invitrogen) and 100 U/mL penicillin-streptomycin (15140122, Invitrogen) during incubation at 37°C in 95% air and 5% CO_2_ humidified incubator. Dispersed A549 cells were transferred to new cell culture dishes for western blotting and agarose spot assay, or culture dishes with glass coverslips for imaging, western blotting, and immunofluorescence.

### Measurement of NBC Activity

A549 cells were attached onto coverslips and loaded with 6 μM 2′,7′-Bis-(2-carboxyethyl)-5-(and-6)-carboxyfluorescein (BCECF-AM, 0061, TEFlabs) along with the same volume of 0.05% pluronic acid (P-3000MP, Invitrogen) for 15 min at room temperature (RT) in the dark. After incubation with the BCECF, the A549 cells were placed on the inverted microscope and perfused with physiological salt solution, as previously described (Ji et al., 2017), for at least 5 min prior to determining the intracellular pH (pH_i_). pH_i_ was determined by measuring BCECF fluorescence using dual excitation wavelengths (495 and 440 nm) and an emission wavelength (530 nm). NBC activity was measured by incubating the cells with a CO_2_-saturated bicarbonate-buffered as described previously (Ji et al., 2017) containing sodium hydrogen exchanger inhibitor 5-(N-ethyl-N-isopropyl)-amiloride (EIPA, 1154-25-2, Sigma Aldrich), followed by acidification with Na^+^-free bicarbonate-buffered medium. Images were obtained with a CCD camera (Q-Imaging) and analyzed with a Meta Fluor system (Molecular Devices). The images were individually normalized by subtracting the background fluorescence signal from the raw background signals.

### Treatment With Small Interfering RNA and DNA Transfection

Small interfering RNA (siRNA) for human SPL was produced using double-promoter pFIV-H1/U6 siRNA cloning and expression vectors (SI111A-1, System Biosciences, Palo Alto, CA, United States), according to manufacturer’s instructions. Purified plasmids contained human siRNA-SPL (sense, 5-AAA GCC AAC CAA GTG TTC AGC ACT TAC TC-3 and anti-sense, 5-AAA AGA GTA AGT GCT GAA CAC TTG GTT GG-3). A549 cells were transfected with 1 μg of siRNA vectors. A549 expressing native SPL were used for evaluating the siRNA-SPL transfection efficacy. The siRNA and plasmid DNA transfection were performed by Lipofectamine 2000 (Invitrogen) and jetPRIME (Polyplus-transfection, France) according to the manufacturer’s protocol, respectively. After a further 4 h of incubation, the medium was changed with new DMEM containing FBS. A549 cells were used for experiments after 48 h of transfection.

### Agarose Spot Assay for Cell Migration

Directional cell migration was examined by performing an agarose spot assay as described previously (Wiggins and Rappoport, 2010; Vinader et al., 2011) with the protocol of the chemotactic invasion assay mildly modified. Briefly, 10 mg of agarose (UltraKem LE, Young Sciences, South Korea) was placed onto a 50 mL conical tube and diluted into 2 mL phosphate-buffered saline (PBS) (pH 7.4) to prepare a 0.5% agarose solution, spotted (four spots per plate) onto six-well plates (Thermo) and allowed to cool for 8 min at 4°C. The 4 × 10^5^ A549 were then plated and allowed to adhere for 4 h before replacing with a medium containing 0.1% FBS (Invitrogen) and 100 U/mL penicillin (Invitrogen) in RPMI. After 24, 48, and 72 h at 37°C, A549 cell images were collected using MetaMorph software (Molecular Devices) with a 10× objective (Olympus).

### Scratch Wound Healing Assay

Scratch wound healing assay to determine the cellular migration ability was performed as previously described (Yarrow et al., 2004; Rodriguez et al., 2005). A549 cells (5 × 10^4^ cells) were cultured in a six-well plate. When the confluence of A549 cell reached approximately 80% and above, scratch wounds were produced with 1000 μL pipet tips in each well. After scratching, the A549 cell debris was removed with pipet and the cell images were obtained after 0, 24, 48, and 72 h of incubation at 37°C and calculated the relative percentage of area in the absence of cell coverage using a microscope (Olympus) with MetaMorph software (Molecular Devices).

### Surface Biotinylation and Western Blotting

The A549 cells were incubated with 1 × lysis buffer (9803, Cell Signaling) containing 150 mM NaCl, 20 mM Tris, 2 mM EDTA, 1% Triton X-100, and a protease inhibitor mixture by passing cell lysates after sonication. The A549 cells were centrifuged at 11,000 × *g* for 15 min at 4°C. Total cellular lysate was collected in supernatant and protein concentration was determined by Bradford assay (5000001, Bio-Rad). To demonstrate the surface expression of proteins, cells were incubated with 0.5 mg/mL EZ-LINK Sulfo-NHS-LC-biotin (21335, Thermo) for 30 min on ice and then followed by previously described (Hwang et al., 2020). Cellular lysates from siRNA-treated cells, transfected cells, or nocodazole-treated cells were also incubated with sample buffer under the same conditions and western blotting was performed as follows. The warmed protein samples (30 μg) were subjected to separation using sodium dodecyl sulfate polyacrylamide gel electrophoresis (SDS-PAGE) and then transferred onto polyvinylidene difluoride membrane (1620177, Bio-Rad) soaked in methanol. The membrane was blocked with 5% non-fat milk solution in Tris-buffered saline (TBS) (150 mM NaCl and 20 mM Tris) and 0.5% Tween-20 (TBS-T) for 1 h. The blocked membrane was incubated with the indicated antibodies overnight at 4°C and washed thrice with TBS-T. Following TBS-T washing, membranes were incubated with horseradish peroxidase-conjugated anti-mouse or anti-rabbit secondary antibodies, associated with each primary antibody, and the signal of protein bands was visualized using the enhanced luminescent solution (32209, Thermo) and X-ray film (Kodak).

### Immunofluorescence and Confocal Imaging

The A549 cells were transferred onto cover glasses and fixed with chilled (−20°C, 10 min) methanol or 4% paraformaldehyde (RT, 10 min). For procedure of paraformaldehyde fixation, cells were permeabilized with 0.5% Triton X-100. Permeabilized cells were treated with 5% goat serum (in PBS) for 1 h at RT to block non-specific sites. Next, the cells were incubated overnight with primary antibodies (1:50∼1:100 dilution factor in 5% goat serum) at 4°C, followed by three washes with incubation buffer (IB, 5% BSA in PBS). To detect bound antibodies, cells were treated with fluorescence-tagged secondary antibodies (1:200 dilution factor), goat immunoglobulin G-tagged with rhodamine (1:50 dilution factor in IB, Jackson ImmunoResearch, anti-mouse: 115-025-072, anti-rabbit: 111-025-144) or fluorescein isothiocyanate (FITC) (1:50 dilution factor in IB, anti-mouse: 115-095-071, anti-rabbit: 111-095-003, Jackson ImmunoResearch) for 1 h at RT. Following incubation, cells were washed with PBS for four times, and the cover glasses were mounted on glass slides using 20 μL Fluoromount-G^TM^ containing 4,6-diamidino-2-phenylindole (DAPI) (17984–24, Electron Microscopy Sciences) and incubated overnight at 4°C in dark place. The slides were analyzed using an LSM 700 Zeiss confocal microscope (Carl Zeiss, Germany) with ZEN software (Carl Zeiss).

### Reverse Transcription-Polymerase Chain Reaction (RT-PCR)

Total RNA was extracted from A549 cells using the Hybrid-RiboEx extraction system (Gentaur, Belgium), according to the manufacturer’s instructions. Amount of isolated RNA was quantified using Spectrophotometer ND-1000 (Thermo Fisher Scientific) and was amplified according to the manufacturer’s protocol of TOPscript^TM^ RT-PCR kit from Enzynomics (Daejeon, South Korea). Primer sequences for human p53 (forward: TGG ATT GGC CAG ACT GCC TTC, reverse: TCT GGA CCT GGG TCT TCA GTG), MMP2 (forward: GCA TCC AGA CTT CCT CAG GC, reverse: CCA TTA GCG CCT CCA TCG TAG), MMP9 (forward: GTA CTC GAC CTG TAC CAG CG, reverse: TTC AGG GCG AGG ACC ATA GA), Ki-67 (forward: AAT TCA GAC TCC ATG TGC CTG AG, reverse: CTT GAC ACA CAC ATT GTC CTC AGC), and GAPDH (forward: CAT GGC ACC GTC AAG GCT GAG, reverse: CTT GGC CAG GGG TGC TAA GC). The amplification protocol of Applied Biosystems (A25742, Thermo) was followed.

### Statistical Analyses

All data from the indicated number of experiments was expressed as the mean ± standard error of the mean (SEM). Statistical differences between mean values obtained from the two or more sample groups were evaluated using Student’s *t*-test. Two independent sample datasets come from distributions with different of two different groups. Significance was statistically determined by analysis of variance (ANOVA) for each experiment (^∗^*p* < 0.05, ^∗∗^*p* < 0.01, ^∗∗∗^*p* < 0.001).

## Results

### SPL Knockdown Promoted Cancer Cell Invasion

**FIGURE 1:**
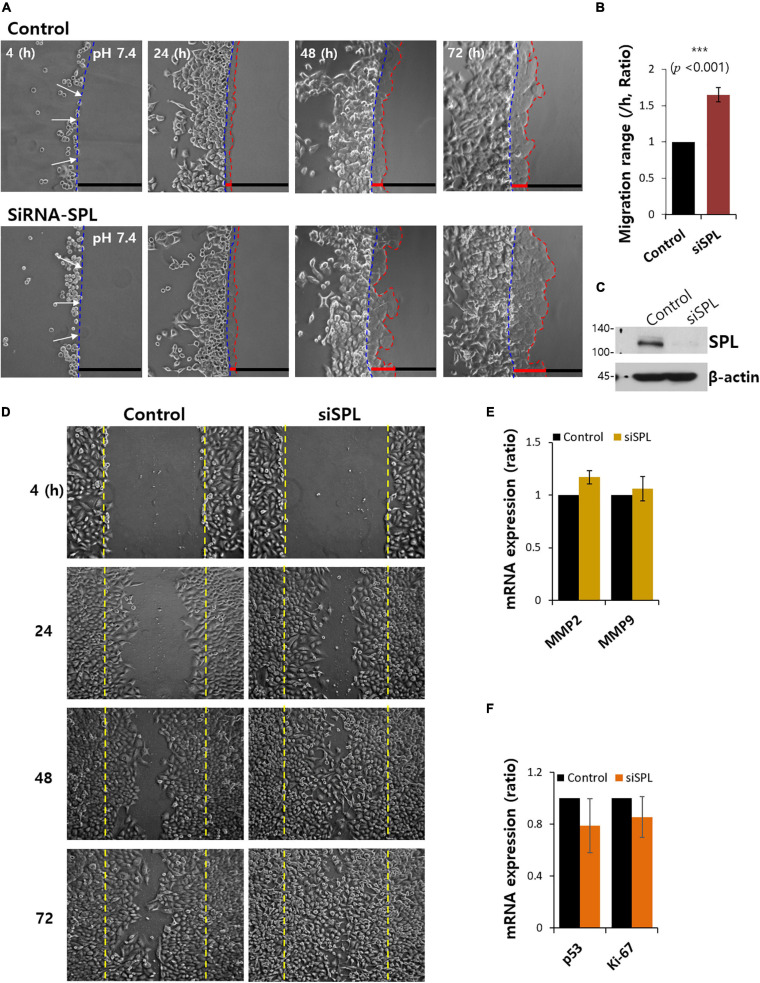
Spinophilin (SPL) knockdown promoted cancer cell invasion. **(A)** Time dependent representative images of A549 cells migrating (4, 24, 48, and 72 h) toward agarose spots containing PBS (pH 7.4) with or without siRNA-SPL (SiSPL). Arrows (white) indicate the direction of migration across the boundary of the agarose spot shown as dotted lines (blue, beginning line). Dotted lines (red) indicate the lineage of cells moved into the agarose spots. **(B)** Analysis of A549 cells migration range per hour in agarose spots. Bars indicate means ± SEM of the number of experiments (*n* = 4). **(C)** Knockdown efficiency of SPL in A549 cells. SPL and β-actin protein expression with or without SiSPL. β-actin antibody was used as a loading control (*n* = 4). **(D)** Scratched wound healing assay of A549 cells migration (4, 24, 48, and 72 h) with and without SiSPL. **(E)** Human MMP2 and MMP9 mRNA expression in the presence of SiSPL at 48 h. **(F)** Human p53 and Ki-67 mRNA expression in the presence of SiSPL at 48 h.

To demonstrate the role of SPL in lung cancer cell migration and invasive motility, the agarose spot migration assay was modified as previously described (Wiggins and Rappoport, 2010). It is well known that cancer cells are exposed to the microenvironment, increasing tissue rigidity during tumor development and progression (Plewes et al., 2000; Paszek et al., 2005). Unlike conventional liquid culture methods, this assay provides matrix-like environments to mimic the cancer microenvironment, allowing the exploration of the function of invadopodia. Invasive A549 cells were compared for motility into agarose spots with and without siRNA-SPL (siSPL) (Figures 1A,B). Control cells were treated with the scramble siRNA vector. On average, about 1.7-fold higher invasive motility was observed in siSPL than in the control (Figure 1B). The siSPL-transfected efficacy was evaluated by western blotting (Figure 1C). To confirm the migratory role of SPL, a scratch wound healing assay was performed. SPL knockdown enhanced the cellular migratory ability (Figure 1D). SPL overexpression also attenuated cellular migration (Supplementary Figures 1A,B). Migratory ability in the presence of siSPL was independent of the expression of matrix metalloproteases, such as MMP2 and MMP9, and cell proliferation markers, such as p53 and Ki-67 (Figures 1E,F). These results indicate that the invasive motility of cancer cells was enhanced by SPL knockdown.

### SPL Modulated the ZO-1 Expression

**FIGURE 2:**
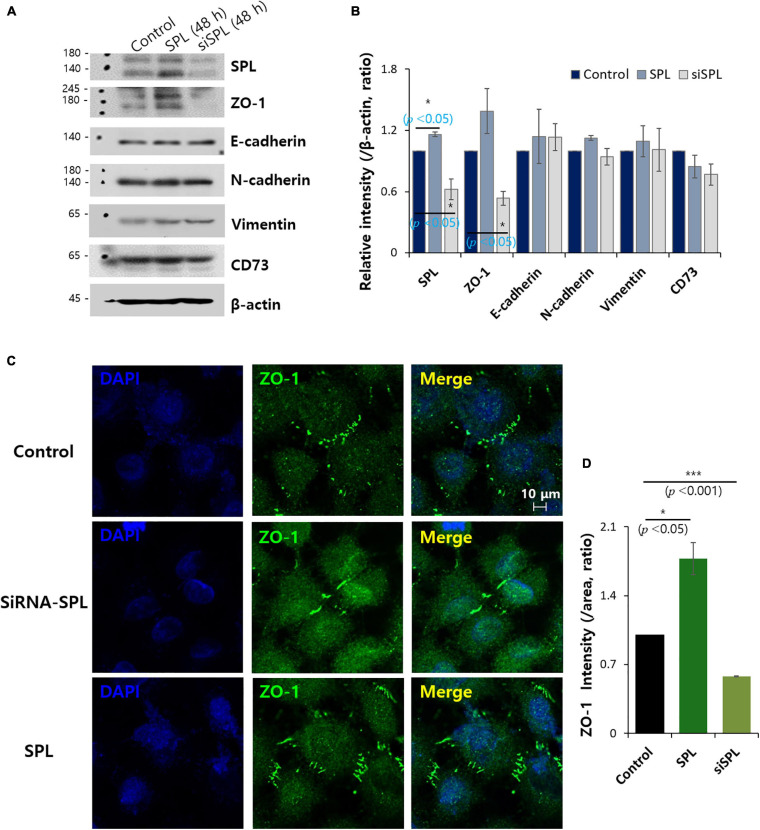
Spinophilin (SPL) modulated ZO-1 expression. **(A)** Protein expression of adhesion protein markers (ZO-1, E-cadherin, N-cadherin, vimentin, and CD73) and SPL in SPL knockdown and overexpression at 48 h. β-actin was used as a loading control. **(B)** Analysis of relative intensity migration range per β-actin. Bars indicate means ± SEM of the number of experiments (*n* = 3). **(C)** Immunostaining of ZO-1 (green) and nucleus (DAPI, blue) in the presence of SiSPL and overexpressed SPL in A549 cells. **(D)** Analysis of ZO-1 intensity. Bars indicate means ± SEM of the number of experiments (*n* = 3).

The modulatory action of SPL on the adhesion markers, ZO-1, E-cadherin, N-cadherin, vimentin, and CD73, was examined in SPL knockdown and overexpression (Figures 2A,B). Knockdown of SPL revealed decreased expression of the tight junction marker ZO-1, whereas showed no effect on other adhesion molecules of epithelial-mesenchymal transition (EMT). To confirm the modulation of ZO-1 expression by SPL, SPL-overexpressed or siSPL-transfected cells were stained with ZO-1. The zipper-like structure of ZO-1 was localized in the cell–cell contact regions (Figure 2C, control panel). SPL knockdown dramatically reduced ZO-1 expression, whereas ZO-1 expression was enhanced at cellular junctions in SPL-overexpressing A549 cells (Figures 2C,D).

### SPL Knockdown Promoted Invadopodium

**FIGURE 3:**
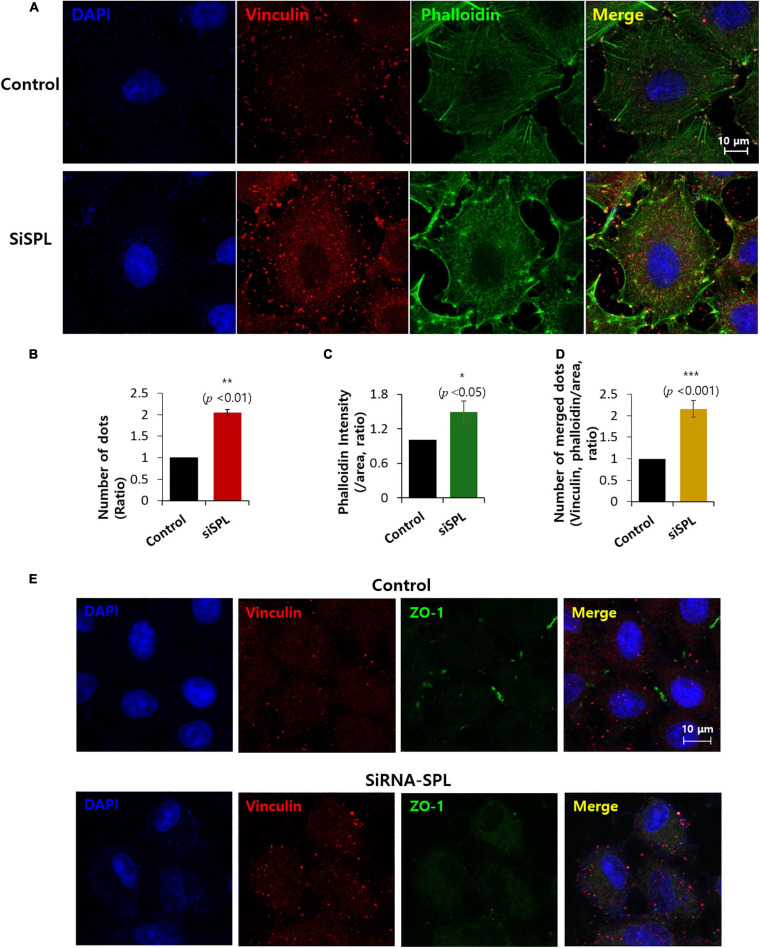
Spinophilin (SPL) knockdown promoted invadopodium. **(A)** Co-immunostaining of phalloidin (green), vinculin (red), and nucleus (DAPI, blue) in the presence of SiSPL at 48 h. **(B)** Analysis of number of vinculin dots in the presence of SiSPL. Bars indicate means ± SEM of the number of experiments (*n* = 4). **(C)** Analysis of phalloidin intensity. Bars indicate means ± SEM of the number of experiments (*n* = 4). **(D)** Analysis of number of merged dots for phalloidin and vinculin. Bars indicate means ± SEM of the number of experiments (*n* = 4). **(E)** Co-immunostaining of vinculin (red), ZO-1 (green), and nucleus (DAPI, blue) in the presence of SiSPL at 48 h.

Invadopodia, actin-rich protrusions of the cell membrane, are common features of cancer cells that lose their adhesive properties and drive cell invasion into the adjacent tissue. This process is called invadopodium (Revach et al., 2015; Eddy et al., 2017). Cells were stained with actin-rich invadopodia marker phalloidin (Wulf et al., 1979; Mazloom-Farsibaf et al., 2021) and the adhesion protein marker vinculin (Citi, 2019) in the presence of siSPL. The number of vinculin dots was enhanced (Figures 3A,B), with the intensity of phalloidin also enhanced in siSPL-treated A549 cells (Figures 3A,C and low magnification images in Supplementary Figure 2). SPL knockdown also enhanced actin-vinculin coupling (Figure 3D). Whereas SPL-overexpressed A549 cells did not modulate the phalloidin intensity (Supplementary Figure 3). We also confirmed that the SPL knockdown reduced ZO-1 expression, whereas vinculin expression was enhanced (Figure 3E).

### SPL Knockdown Enhanced Sodium/Bicarbonate Cotransporter Activity

**FIGURE 4:**
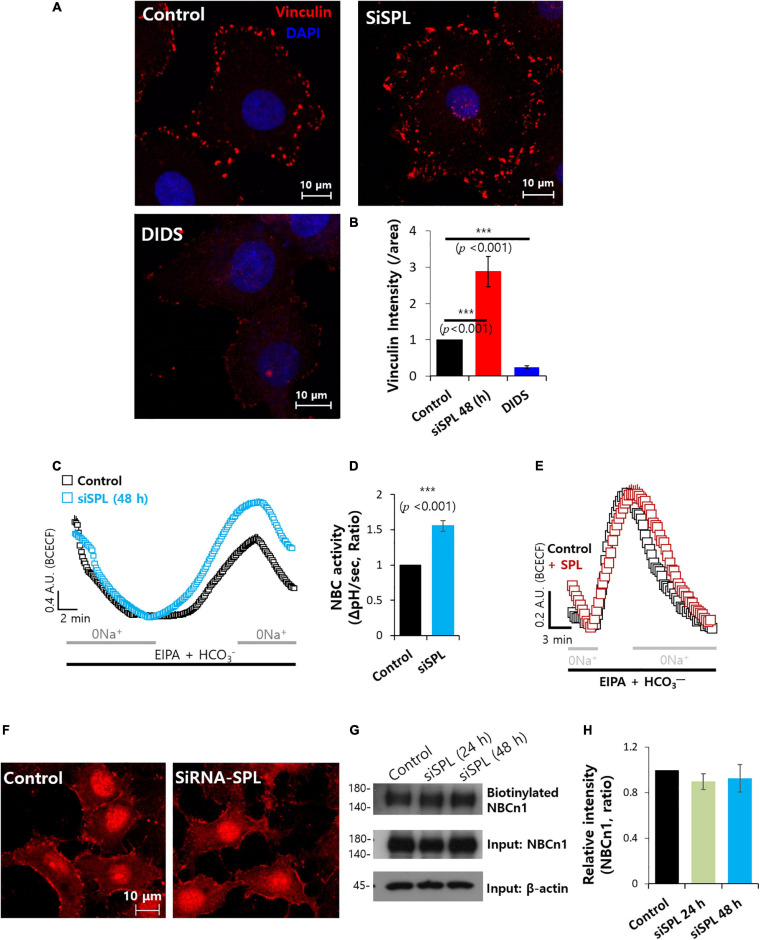
Spinophilin (SPL) knockdown enhanced sodium/bicarbonate cotransporter activity. **(A)** Immunostaining of vinculin (red) and nucleus (DAPI, blue) in the presence of SiSPL or DIDS at 48 h. **(B)** Analysis of vinculin intensity. Bars indicate means ± SEM of the number of experiments (*n* = 4). **(C)** NBC activity of A549 cells with (blue open square) or without (black open square) SiSPL at 48 h. Averaged traces and error bars were represented. **(D)** Bars indicate means ± SEM of the number of experiments (*n* = 5). **(E)** NBC activity of A549 cells with (red open square) or without (black open square) SPL. Averaged traces and error bars were represented. **(F)** Immunostaining of NBCn1 (red) in the presence of SiSPL at 48 h. **(G)** Surface expression of NBCn1 of SiSPL in A549 cells at 48 h. Input β-actin and NBCn1 blots were used as loading controls. **(H)** Analysis of NBCn1 intensity. Bars indicate means ± SEM of the number of experiments (*n* = 4).

Various evidence indicates that enhanced bicarbonate transporter activity has been associated with increased migratory ability, resulting from metabolic adaptation of extra- and intracellular pH dynamics (White et al., 2017). The verification of sensitivity of DIDS represents the extent of the bicarbonate transporter contribution (Mastrocola et al., 1991). DIDS-treated cells showed dramatically reduced vinculin expression, whereas knockdown of SPL enhanced vinculin expression (Figures 4A,B). Overexpression of SPL mildly attenuated vinculin intensity (Supplementary Figure 4). To obtain evidence for direct interaction between SPL and the bicarbonate transporter, we examined the relationship between the knockdown of SPL and sodium/bicarbonate cotransporter (NBC) activity. Knockdown of SPL enhanced NBC activity (Figures 4C,D). Whereas overexpression of SPL did not modulate NBC activity (Figure 4E). Protein expression and membrane localization of NBCn1 were not affected by SPL knockdown (Figures 4F–H). These results addressed that attenuation of SPL enhanced NBC activity and was independent expression of NBCn1.

### Knockdown of SPL Modulated Tubulin Structure

**FIGURE 5:**
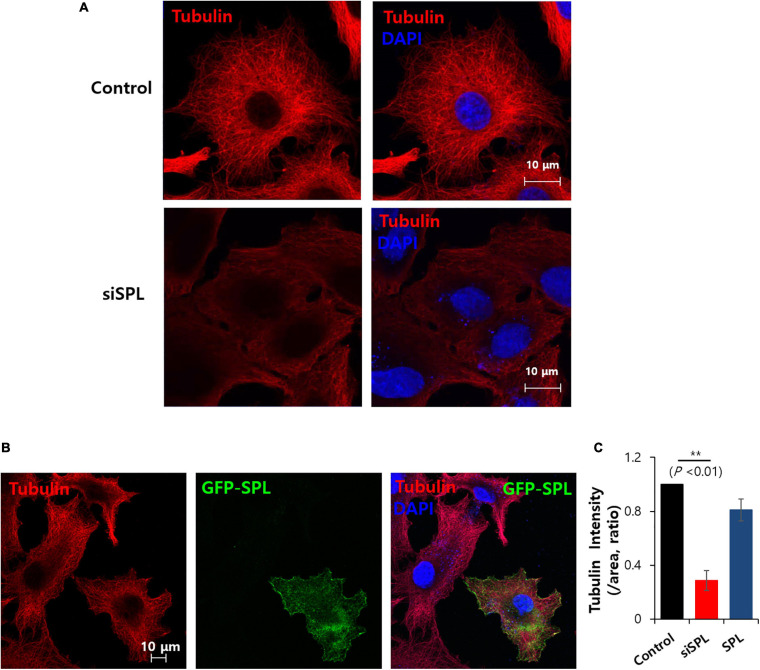
Spinophilin (SPL) knockdown modulated tubulin structure. **(A)** Immunostaining of tubulin (red) and nucleus (DAPI, blue) in the presence of SiSPL at 48 h. **(B)** Immunostaining of tubulin (red), SPL (green), and nucleus (DAPI, blue) in the presence of GFP-tagged SPL. **(C)** Analysis of tubulin intensity. Bars indicate means ± SEM of the number of experiments (*n* = 4).

Spinophilin is associated with microtubule bundling, which is associated with cytoskeletal networks (Bielas et al., 2007). This study verified the tubulin structure in the presence of siRNA-SPL. Tubulin intensity was reduced and diffused staining of tubulin was revealed by the knockdown of SPL (Figures 5A,C). Whereas overexpression of SPL did not modulate tubulin intensity and structure (Figures 5B,C). These results addressed the knockdown of the SPL-modulated tubulin expression.

### Microtubule Destabilization With Nocodazole Reduced NBC Activity

**FIGURE 6:**
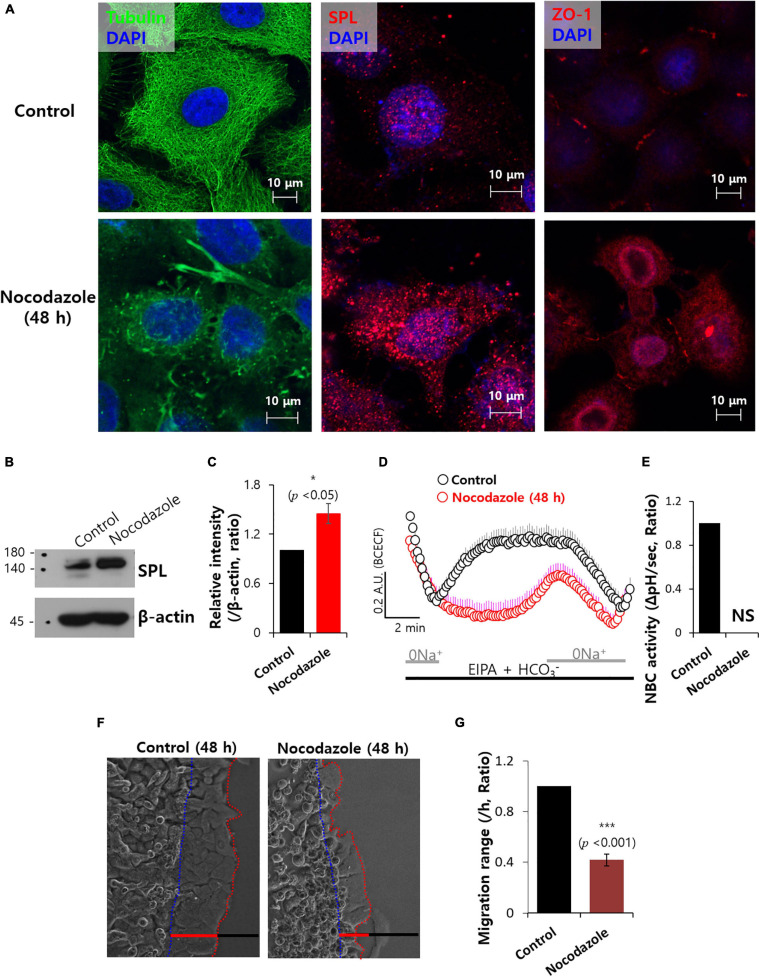
Microtubule destabilization with nocodazole reduced NBC activity. **(A)** Immunostaining of tubulin (green), SPL and ZO-1 (red), and nucleus (DAPI, blue) in the presence of 100 μM nocodazole at 48 h. **(B)** SPL expression of 100 μM nocodazole at 48 h. β-actin was used as a loading control. **(C)** Bars indicate means ± SEM of the number of experiments (*n* = 4). **(D)** NBC activity of A549 cells with (red open circles) and without (control, black open circles) 100 μM nocodazole at 48 h. Averaged traces and error bars were represented. **(E)** Bars indicate means ± SEM of the number of experiments (*n* = 4). NS: not stipulated. **(F)** Time dependent representative images of A549 cells migrating at 48 h toward agarose spots containing PBS (pH 7.4) with and without 100 μM nocodazole. Dotted lines (red) indicate the lineage of cells moved into the agarose spots (blue dotted line) **(G)** Analysis of A549 cells migration range per hour. Bars indicate means ± SEM of the number of experiments (*n* = 4).

Attenuated SPL affected the migration of tubulin structures. To address the relationship between SPL and tubular structure, microtubule polymerization inhibitor nocodazole was administrated, which mediates destabilization of microtubules (Alves-Silva et al., 2017). Treatment with nocodazole revealed tubulin destabilization and enhanced SPL and ZO-1 expression (Figure 6A). Enhanced SPL expression in the presence of nocodazole treatment was also observed (Figures 6B,C). However, nocodazole treatment reduced NBC activity (Figures 6D,E). Thus, migration of A549 cancer cells was also inhibited by nocodazole (Figures 6F,G). To confirm the effect of structural stabilization on NBC activity, cells were stained with tubulin in the presence of actin polymerization destabilizer cytochalasin D (Dietzel et al., 2013). As shown in Figure 6, cytochalasin D treatment revealed the structural modification of tubulin (Supplementary Figure 5A) and reduced NBC activity (Supplementary Figures 5B,C). These results suggest that structural stabilization is critical for maintaining the NBC activity to migrate.

## Discussion

To identify the promiscuous molecular role of SPL, here, we determined the role of SPL in the modulation of the junctional network to perform the concerted interplay with ZO-1 and the cellular migration in lung cancer cells A549. SPL expression modulated the invadopodium-associated protein vinculin to regulate cancer cell migration. SPL knockdown enhanced NBC activity to provide motility. SPL knockdown may modulate the rigidity of cell motile mass or remodel tight junctions, which provide favorable circumstances for migratory machinery such as loosened tight junctions.

The three major steps in the metastatic cascade of cancer cells are as follows: (1) low cell–cell interaction, (2) invasion of stroma and vasculature, and (3) adhesion to the endothelium (Stock and Schwab, 2015). Cancer cells transform mesenchymal feature-positive cells that invade extracellular matrix (Pastushenko and Blanpain, 2019). The EMT is a fundamental process to acquire mesenchymal features. In this study, although attenuation of SPL protein did not modulate expression of EMT proteins, reduced tight junctional protein ZO-1 and enhanced NBC activity were observed. We previously reported that SPL did not modulate NBC activity (Lee et al., 2018). NBCn1 has been reported to function as the migratory machinery in cancer cells (Hwang et al., 2020). Notably, SPL knockdown led to enhanced NBC activity, as shown in Figures 4C,D. At the beginning of the experiments, the focus was on NBCn1 as the migratory machinery. Not only the migratory role but also the pH-regulating role of transporters is important in the formation of invadopodia through the modulation of actin dynamics (Stock and Schwab, 2015). Interestingly, the expression status of SPL modulated the NBC activity, even though SPL has a classical adaptive role in synapses (Allen et al., 2006) and there was no molecular interaction between SPL and NBC (Ji et al., 2017). Preferably, the consistency of the tubular structure influenced NBC activity. Stability of tight junction also should be considered in late-limiting component to regulate NBC activity. Modulated NBC activity provides acidic extracellular circumstances with view of pH regulation because of bicarbonate influx. In addition, ion movement by this NBC activity provides ion and water flux into the cells, which offers migratory machinery with view of metastatic process. Since the sodium hydrogen exchanger NHE is known to be a major transporter in various tumors (Reshkin et al., 2000; Chiang et al., 2008), NBCn1 also contributes to breast and lung cancer (Boedtkjer et al., 2013; Hwang et al., 2020). Experimental evidence in this study using the NHE inhibitor EIPA revealed substantial NBC involvement. With the sequential process, it could not however be concluded whether initial signals by SPL-mediated tubular structural changes affected sequential transporter involvement or occurred simultaneously.

Accumulating evidence has addressed that the expression level of SPL is correlated with proliferative activity (Carnero, 2012). It has been also reported that SPL reduction correlates with malignant grade of tumor and proliferative marker p53 mutation (Molina-Pinelo et al., 2011). Reduced expression of SPL enhanced oncogenic features of cells regardless absence or mutation of p53 (Aigelsreiter et al., 2013). As shown in Figure 1F, knockdown of SPL did not modulate proliferative markers, p53 and Ki-67. We hypothesized that the siRNA-based knockdown *in vitro* approach was performed in the beginning of depleted scaffolding protein SPL. The transfected A549 showed a significant increase of cellular migration when mRNA of SPL was silenced. These data support the hypothesis that a reduced expression of SPL might turn on the start-up module through debacle of tight junction for migratory ability and acid extrusion for invasive ability. Although EMT is not a simple binary process, this feature would be a beginning module before EMT.

Tight junction mainly composes barrier formation and regulates epithelial cell permeability [reviewed in Anderson and Van Itallie (2009); Krug et al. (2014)]. Barrier function of tight junction is related to various human diseases such as inflammatory bowel disease (Zeissig et al., 2007), multiple sclerosis (Forster et al., 2007), and cystic fibrosis (Coyne et al., 2002) including cancer cell invasiveness. The structural consistency of tight junctions is considered an inhibitory factor in cancer cell migration and metastatic processes in various tissues including gastric and liver cancer (Wei et al., 2019; Zhang et al., 2019). Fundamental role of barrier, tight junction plays a central role in modulation of cellular stiffness, a critical cellular characteristic that changes during cellular migratory and adhesive process (Luo et al., 2016; Citi, 2019). In addition to interaction of diverse intracellular signaling proteins such as Raf1 and Rho with tight junction induces various signaling process including cell polarity, differentiation, growth, and proliferation (Schneeberger and Lynch, 2004; Wang et al., 2005; Terry et al., 2010).

**FIGURE 7:**
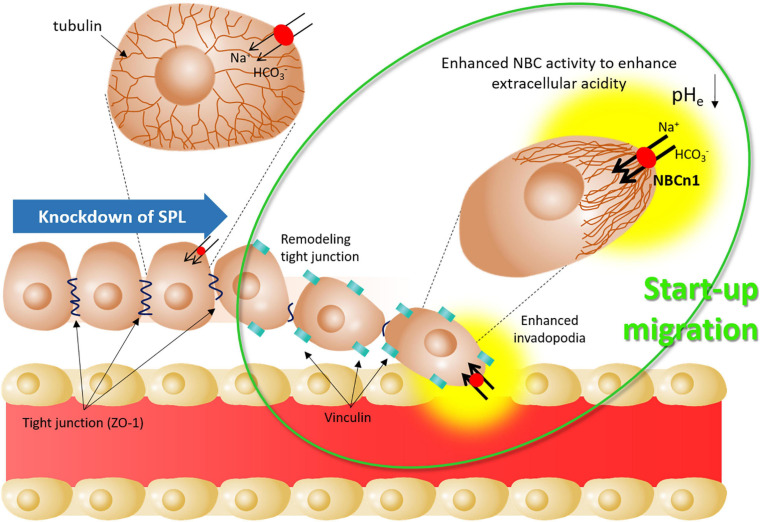
Schematic illustration of start-up migration through SPL knockdown. Attenuated SPL provides mode of start-up migration, including remodeling of tight junctions by reduced ZO-1 expression, enhanced invadopodia by enhanced vinculin, and enhanced NBC activity to enhance extracellular acidity. SPL: spinophilin, NBCn1: electro-neutral form of sodium bicarbonate cotransporter 1, pH_e_: extracellular pH.

This study revealed that SPL-mediated junctional modulation and tubular stability affected bicarbonate transporter activity in lung cancer cells. Attenuation of SPL provides activates start-up migration through the remodeling of tight junctions, enhancement of NBC activity to promote acid extrusion, and formation of invadopodia (Figure 7). The junctional modulatory function of SPL and start-up migratory modification revealed here would support fundamental research and develop the initial target against lung cancer cell migration.

## Data Availability Statement

The original contributions presented in the study are included in the article/Supplementary Material, further inquiries can be directed to the corresponding author/s.

## Ethics Statement

This study does not include any studies with human participants or animals performed by any of the authors.

## Author Contributions

JH, PC-WL, and DS conceptualized and designed the study. SH, PC-WL, and JH prepared the data, performed the analysis, and revised the manuscript critically for intellectual content. JH and PC-WL acquired the funding resource. JH and DS approved the final version of the manuscript. All authors contributed to the article and approved the submitted version.

## Conflict of Interest

The authors declare that the research was conducted in the absence of any commercial or financial relationships that could be construed as a potential conflict of interest.
