# Supplementary material for: Modulated Start-Up Mode of Cancer Cell Migration Through Spinophilin-Tubular Networks
Source: Front Cell Dev Biol. 2021 Mar 9;9:652791. doi: 10.3389/fcell.2021.652791 (PMC7985070; doi:10.3389/fcell.2021.652791)
Supplement: Supplementary file 1 [file Presentation_1.pdf]

# Modulated start-up mode of cancer cell migration through spinophilin-tubular networks

Soyoung Hwang[1], Peter Chang-Whan Lee[2], Dong Min Shin[3†], and Jeong Hee Hong[1,4†]

## Supplementary Figures

## Supplementary Figure S1. Hwang et al.

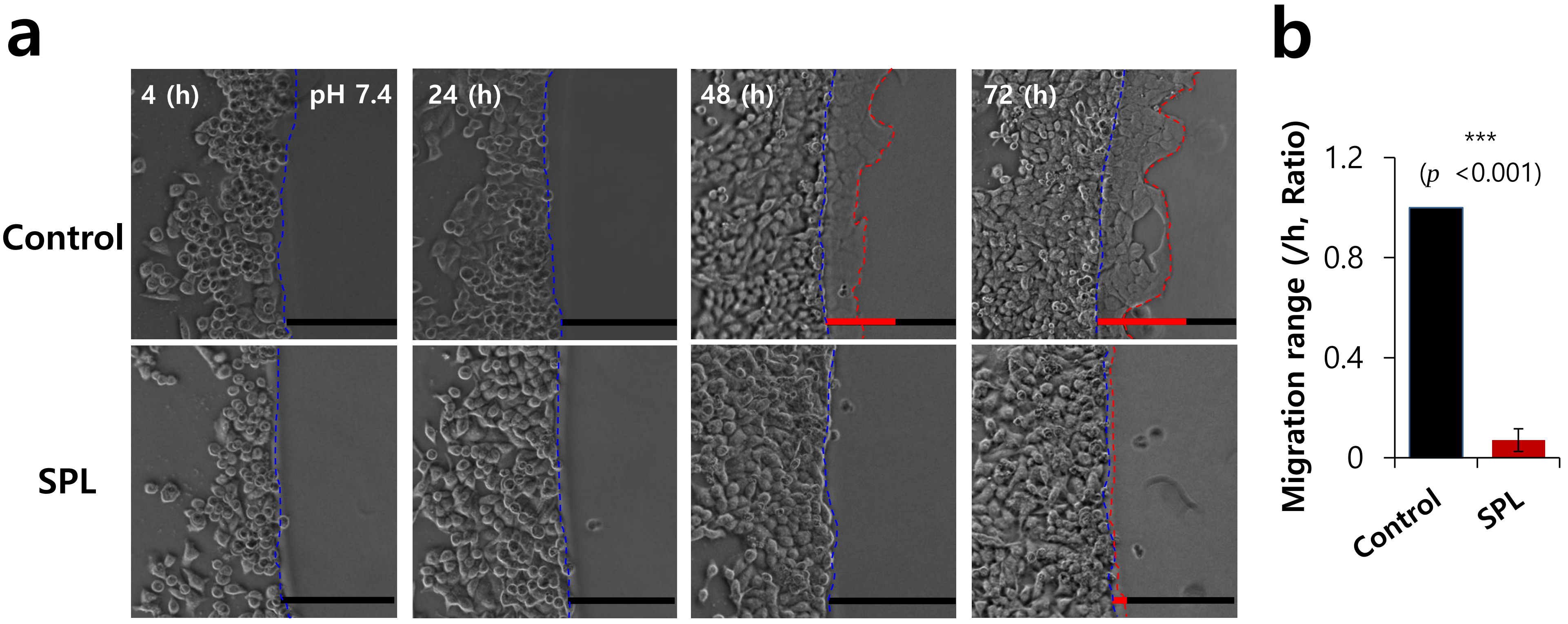


**Figure S1. Overexpression of SPL inhibits cancer cell migration**

**(a)** Time dependent images of A549 cells migrating (4, 24, 48, and 72 h) towards agarose spots containing PBS (pH 7.4) with or without SPL overexpression. The direction of migration across the boundary of the agarose spot shown as dotted lines (blue). The dotted lines (red) indicate the lineage of cells moved into the spots. **(b)** Analysis of migration range per hour. Bars indicate the means ± SEM of the number of experiments (n = 4).

# Supplementary Figure S2. Hwang et al.

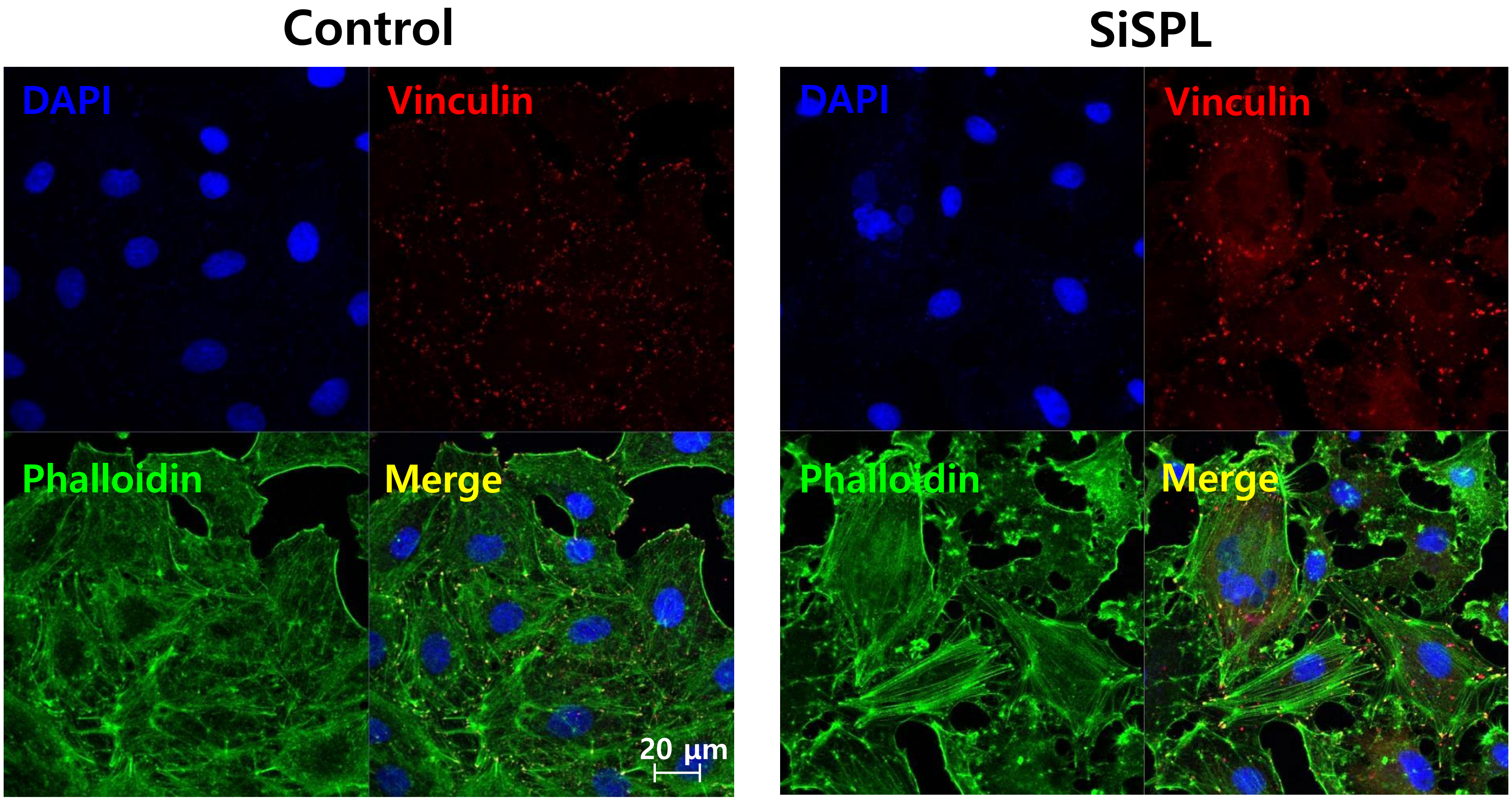


**Figure S2. Knockdown of SPL enhances vinculin expression**

Immunostaining of vinculin (red), phalloidin (green), and nucleus (DAPI, blue) in the presence of SiSPL at 48 h.

## Supplementary Figure S3. Hwang et al.

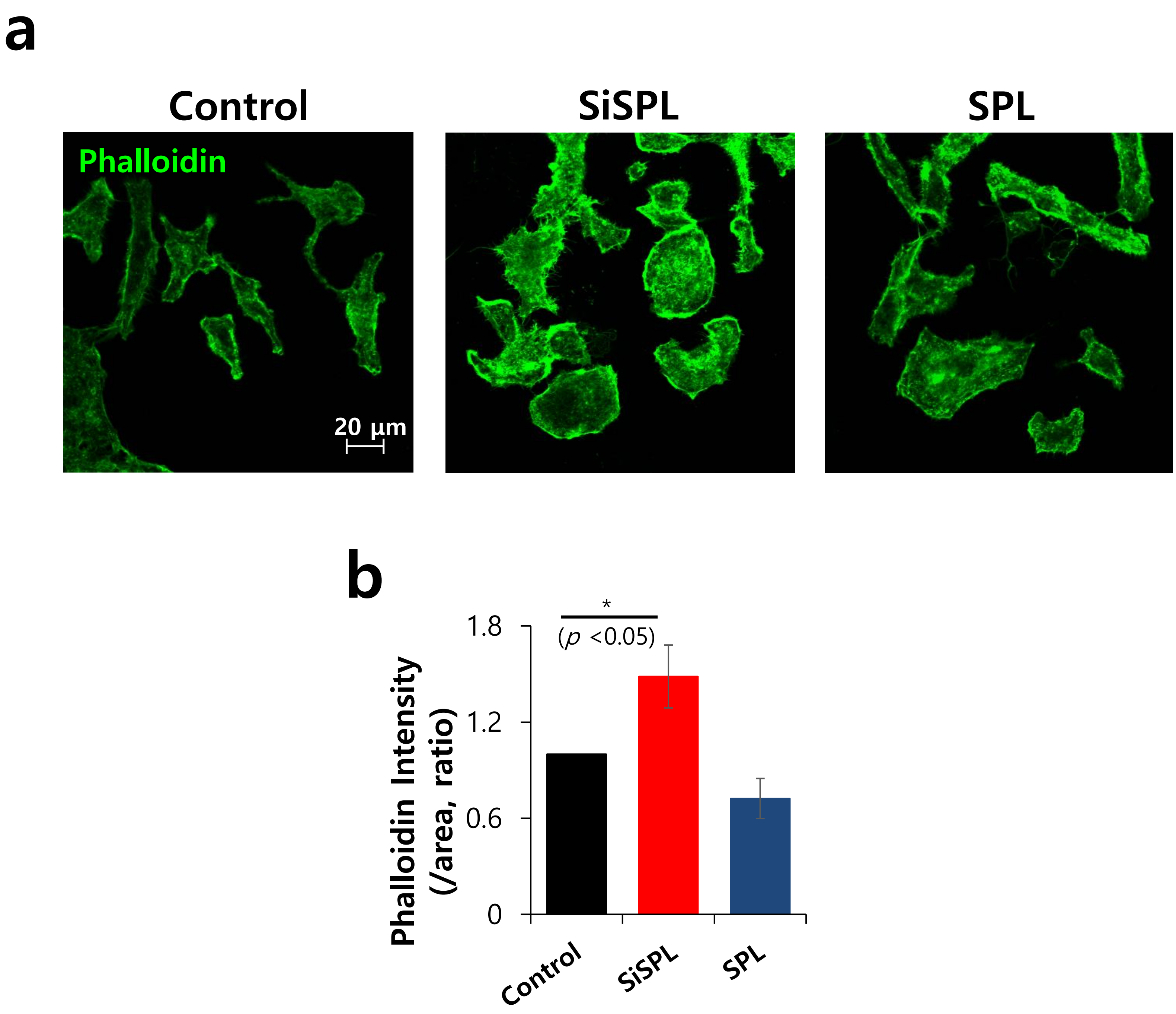


**Figure S3. Knockdown of SPL enhances phalloidin expression, whereas overexpressed SPL did not modulate phalloidin expression.**

**(a)** Immunostaining of phalloidin (green) in the presence of SiSPL at 48 h and in overexpressed SPL. **(b)** Bars indicate means ± SEM of the number of experiments (n = 5).

# Supplementary Figure S4. Hwang et al.

a

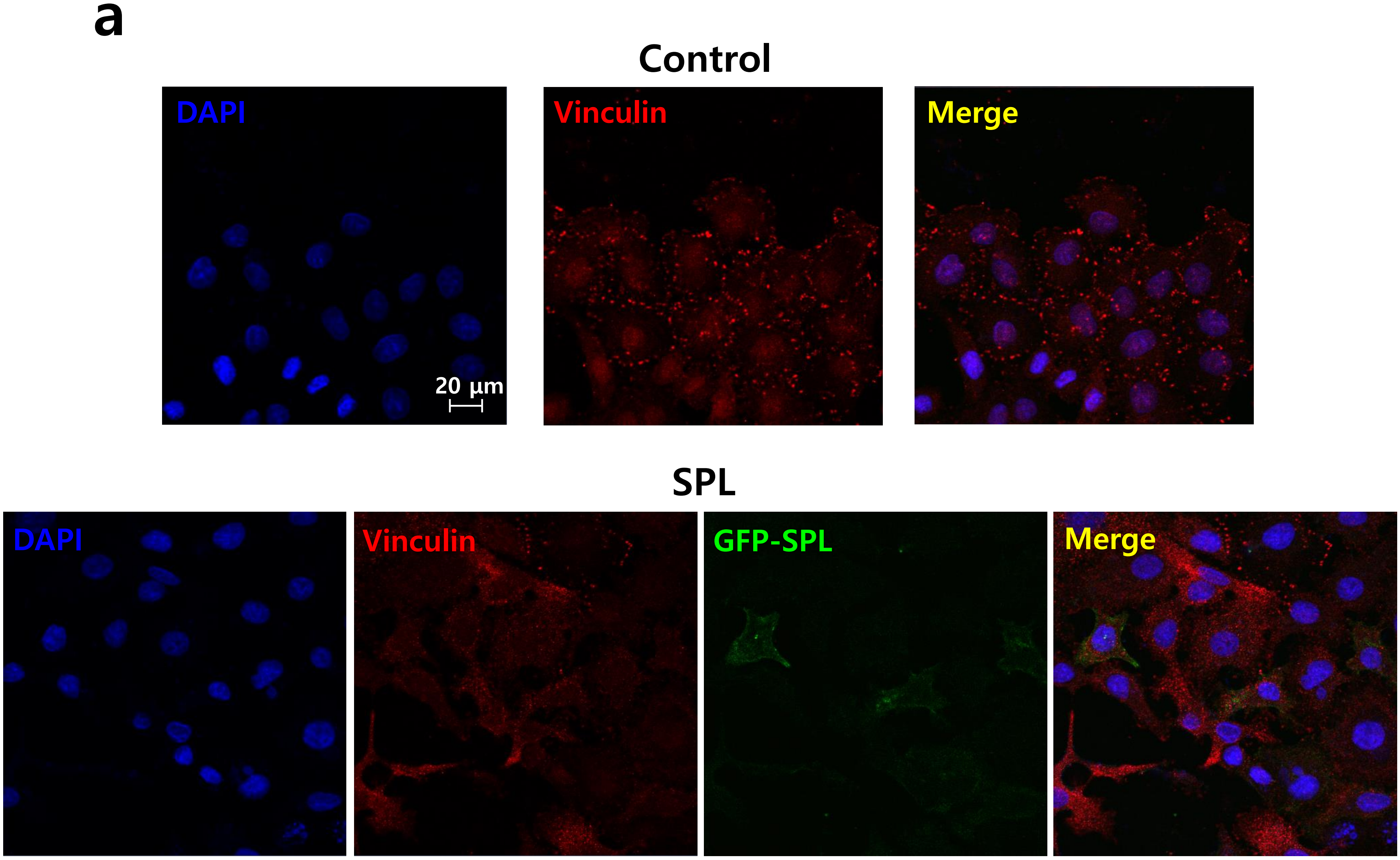


b

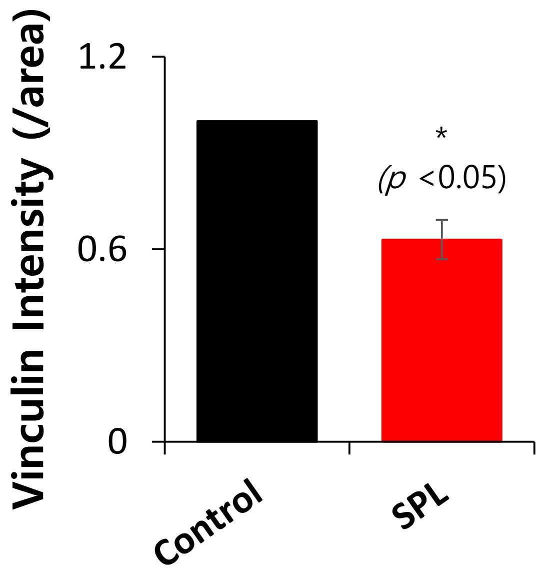


**Figure S4. Overexpressed SPL reduces vinculin expression**

**(a)** Immunostaining of vinculin (red), SPL (green), and nucleus (DAPI, blue). A549 cells were overexpressed GFP-tagged SPL. **(b)** Bars indicate means ± SEM of the number of experiments (n = 5).

# Supplementary Figure S5. Hwang et al.

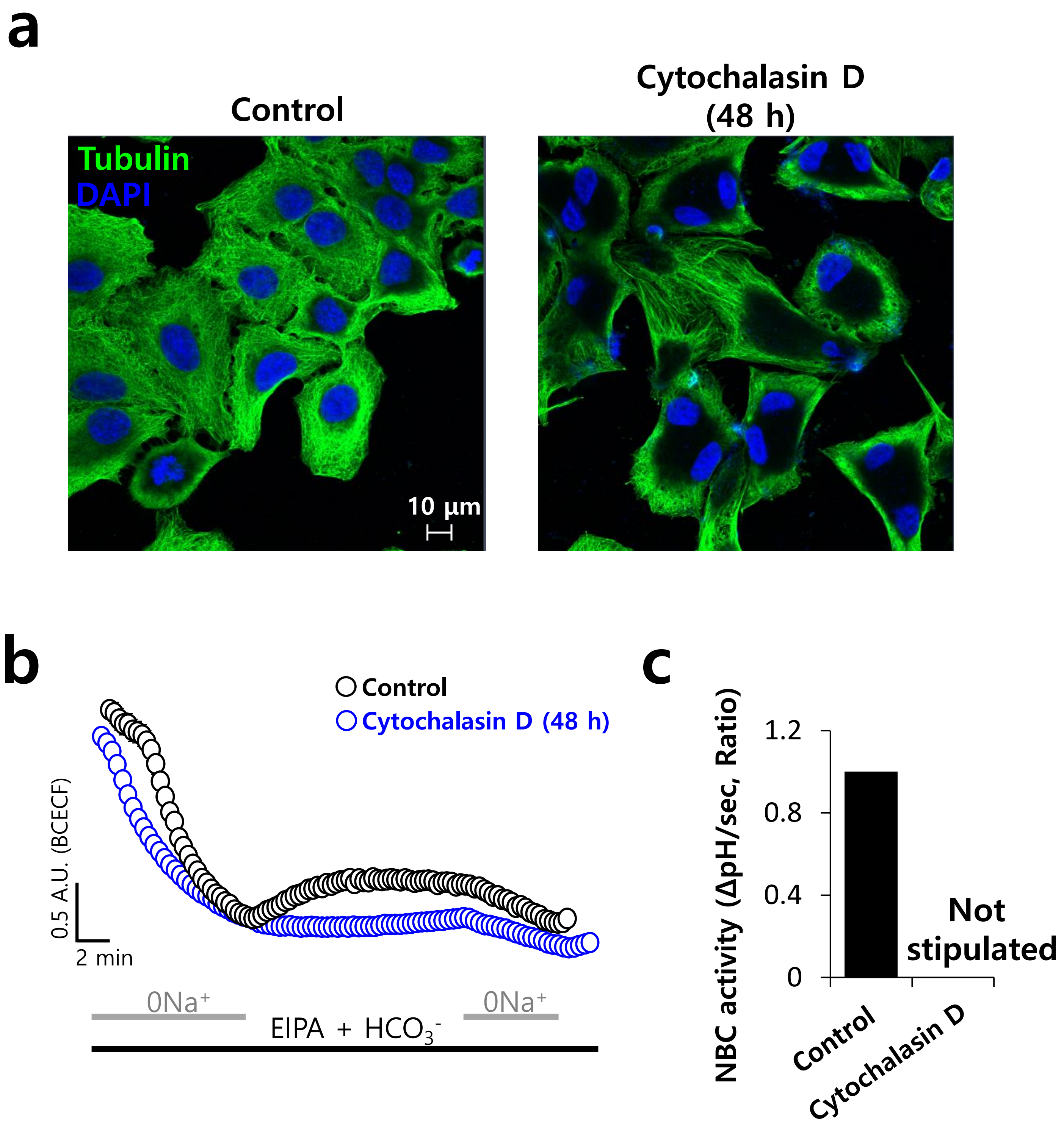


**Figure S5. Actin destabilizer cytochalasin D reduces NBC activity**

**(a)** Immunostaining of tubulin (green) and nucleus (DAPI, blue) in the presence of 1.67 μM cytochalasin D at 48 h. **(b)** NBC activity of A549 cells with (blue open circles) and without (black open circles) 1.67 μM cytochalasin D at 48 h. Averaged traces were represented. **(c)** Bars indicate means ± SEM of the number of experiments (n = 4).
