# Supplementary material for: Modulated Start-Up Mode of Cancer Cell Migration Through Spinophilin-Tubular Networks
Source: Front Cell Dev Biol. 2021 Mar 9;9:652791. doi: 10.3389/fcell.2021.652791 (PMC7985070; doi:10.3389/fcell.2021.652791)
Supplement: Supplementary file 2 [file Data_Sheet_1.pdf]

Modulated start-up mode of cancer cell migration through spinophilin-tubular networks

Soyoung Hwang[1], Peter Chang-Whan Lee[2], Dong Min Shin[3†], and Jeong Hee Hong[1,4†]

Department of Physiology, College of Medicine, Department of Health Sciences and Technology, GAIHST, Gachon University, 155 Getbeolro, Yeonsu-gu, Incheon, 21999, South Korea

Running title: Spinophilin modulates cell migration

Key words: spinophilin, migration, tight junction, tubular network, bicarbonate transporter

[†] Correspondence to JHH (minicleo@gachon.ac.kr) and DMS (dmshin@yuhs.ac)

Authors:

Soyoung Hwang
[1]Department of Physiology, College of Medicine, Gachon University, 155 Getbeolro, Yeonsu-gu, Incheon, 21999, South Korea
Phone: +82-32-899-6434
E-mail:snrntlwy1004@gmail.com

Peter Chang-Whan Lee
[2]Department of Biomedical Sciences, University of Ulsan College of Medicine, Asan Medical Center, Seoul 05505, Korea.
Phone: +82-2-3010-2799
E-mail: pclee@amc.seoul.kr

[†]Dong Min Shin
[3]Department of Oral Biology, Yonsei University College of Dentistry, Seoul 03722, Republic of Korea
Phone: +82-2-2228-3051
E-mail: dmshin@yuhs.ac

[†]Jeong Hee Hong, PhD,
[1]Department of Physiology, College of Medicine, Gachon University, 155 Getbeolro, Yeonsu-gu, Incheon, 21999, South Korea
[4]Department of Health Sciences and Technology, GAIHST, Lee Gil Ya Cancer and Diabetes Institute, 155 Getbeolro, Yeonsu-gu, Incheon, 21999, South Korea
Phone: +82-32-899-6682
Fax: +82-32-899-6039
E-mail: minicleo@gachon.ac.kr

Figure 1. Hwang et al.

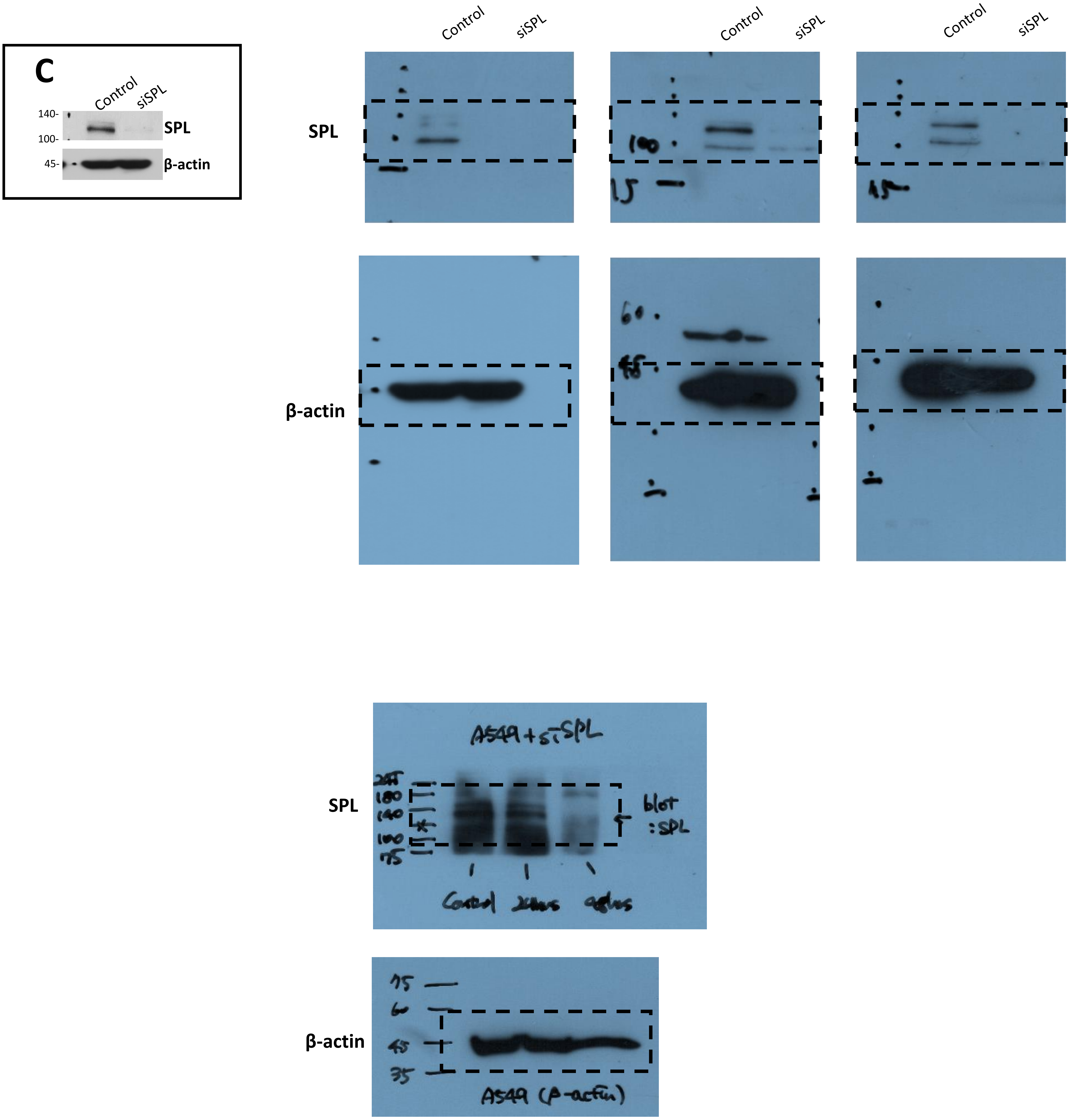

Figure 1. (C) Knockdown efficiency of SPL in A549 cells. The protein expression of SPL and β-actin with and without SiSPL. The β-actin antibody was used as a loading control.

Figure 2. Hwang et al.

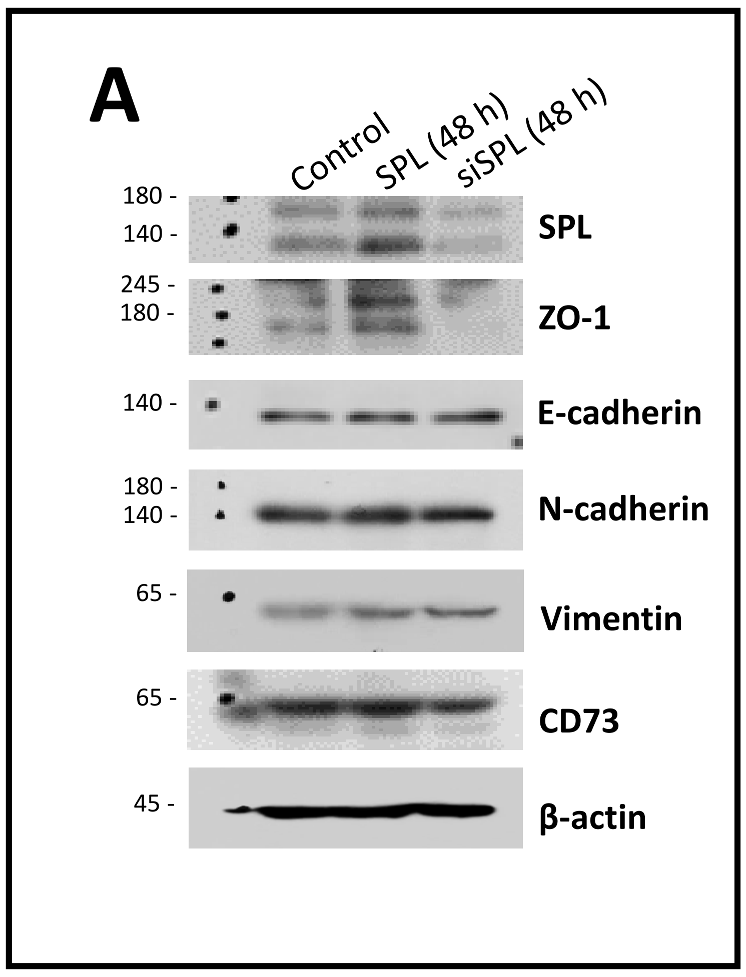

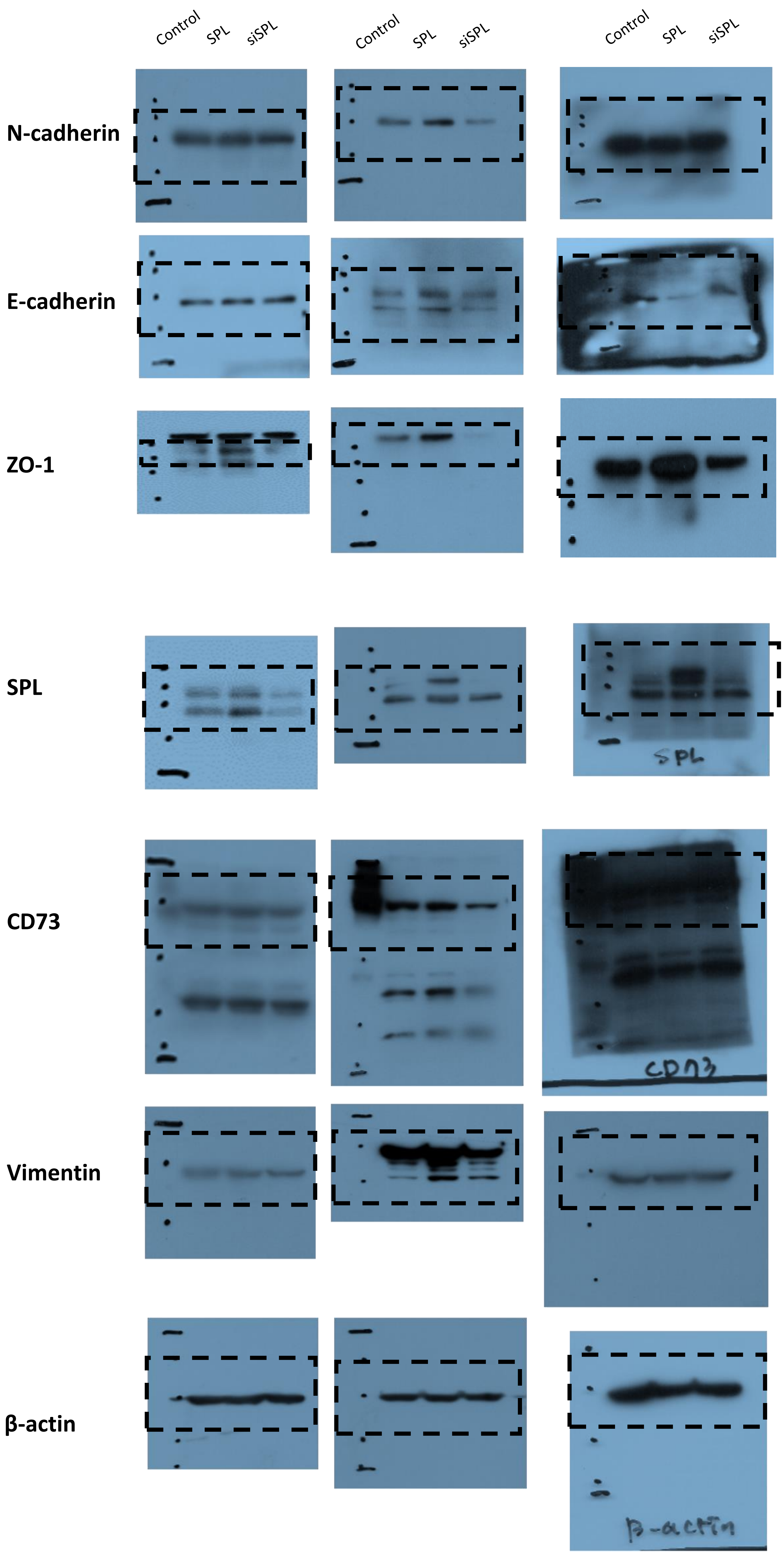

Figure 2. (A) Protein expression of EMT markers in A549 cells in the presence of SiRNA-SPL (SiSPL). β-actin were used as loading controls.

Figure 4. Hwang et al.

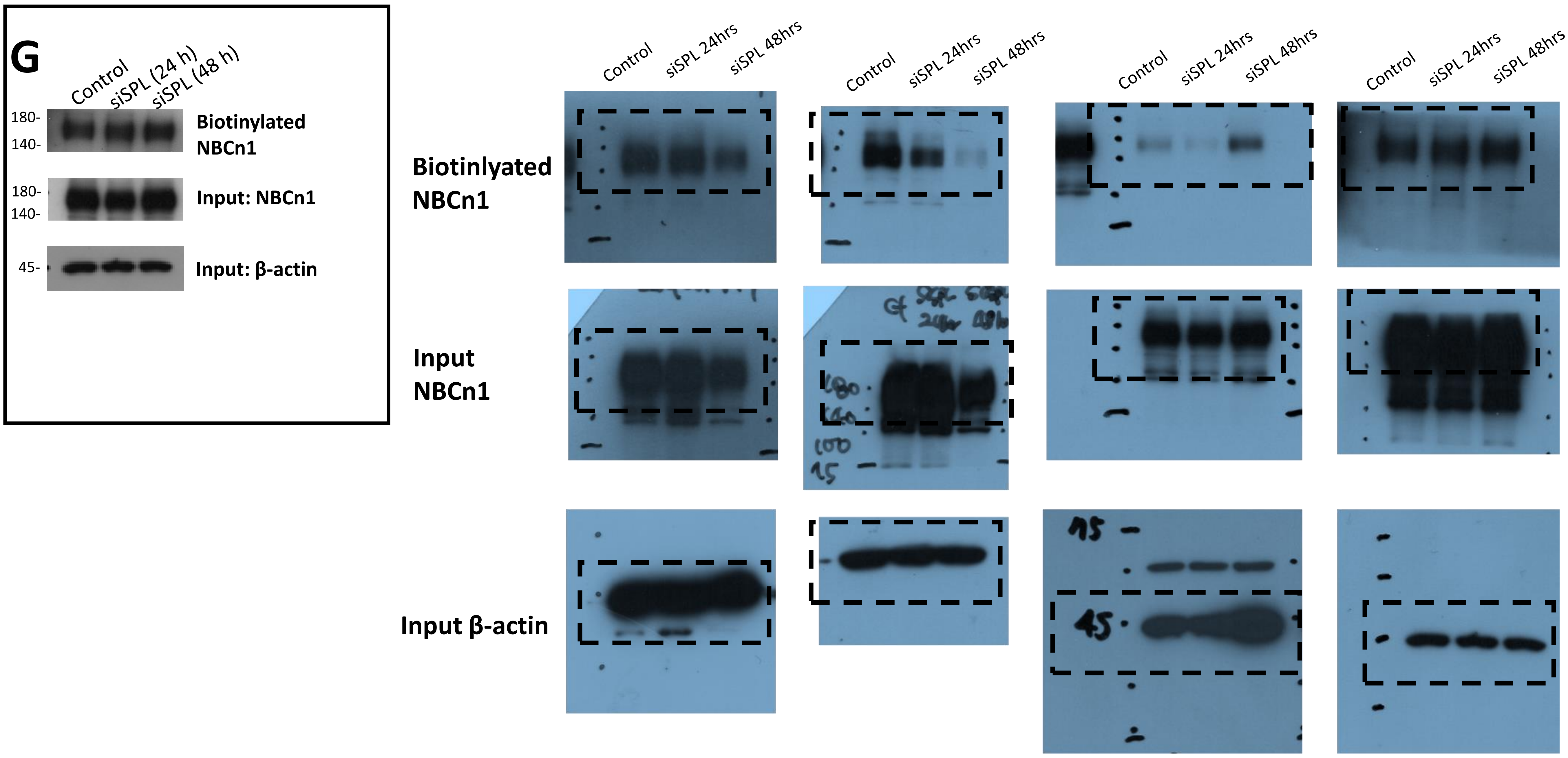

Figure 4. (G) Surface expression of NBCn1 in the presence of siSPL in A549 cells. Input β-actin, input NBCn1, and input SPL blots were used as loading controls.

Figure 6. Hwang et al.

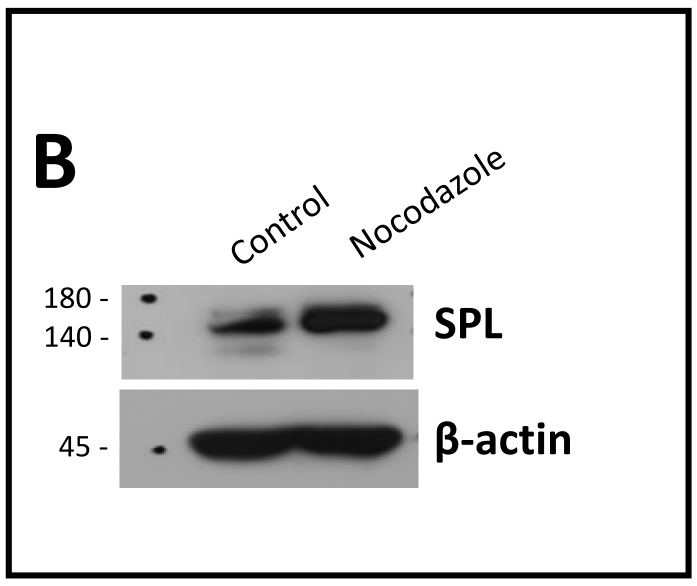

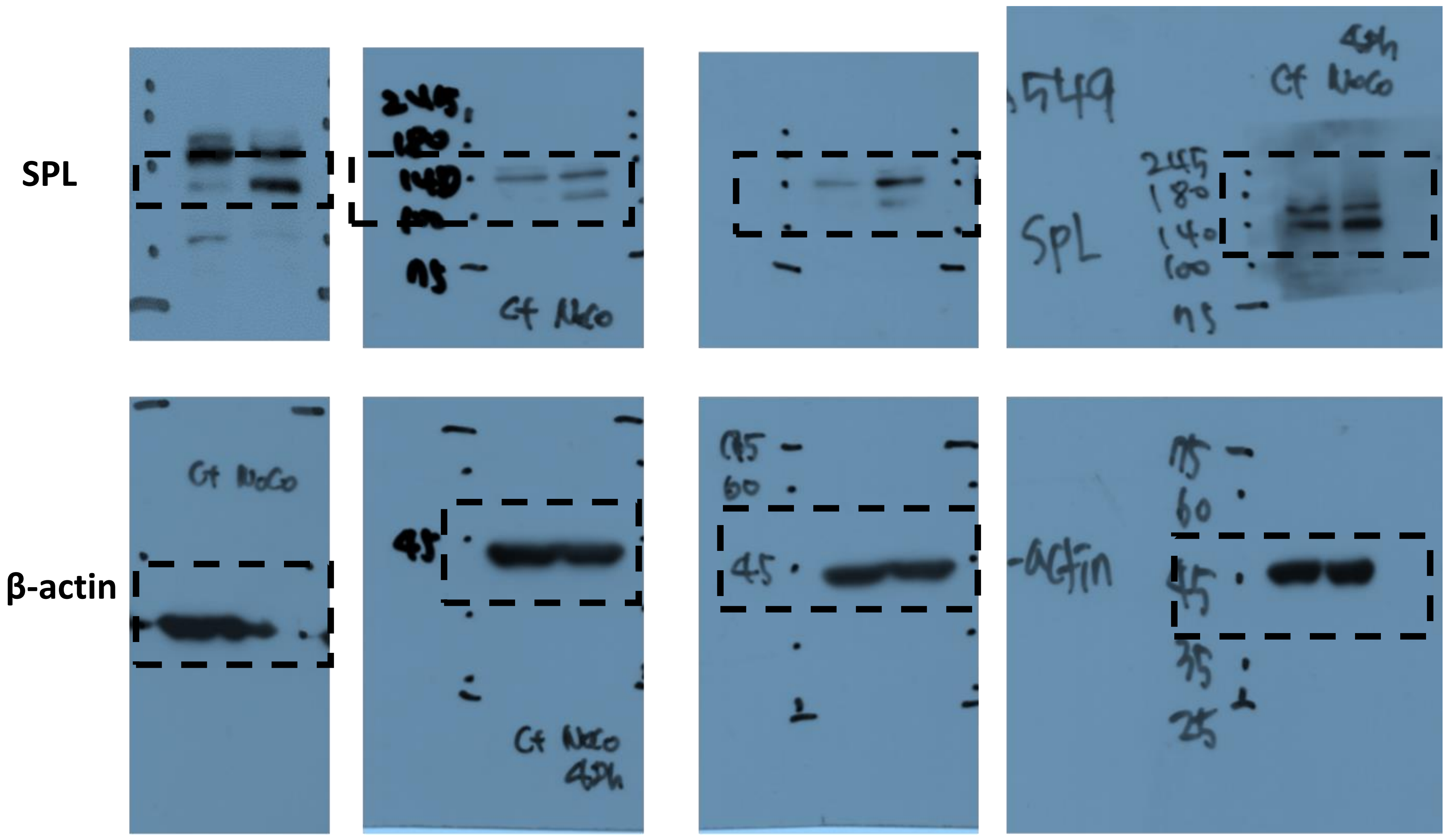

Figure 6. (B) Protein expression of SPL in A549 cells in the presence of 100 μM Nocodazole in 48 hr. β-actin were used as loading controls
